# Gender-Based Differences in X Usage Among Orthopedic Surgeons at Top-Ranked US Hospitals: Cross-Sectional Analysis

**DOI:** 10.2196/69366

**Published:** 2025-06-06

**Authors:** Jordan O Gasho, Daniel G Tobert, Katelyn M Atkins

**Affiliations:** 1 Keck School of Medicine of USC Los Angeles, CA United States; 2 Massachusetts General Hospital Boston, MA United States; 3 Cedars-Sinai Medical Center Los Angeles, CA United States

**Keywords:** social media, X (Twitter), gender disparities, orthopedic surgery, physician workforce disparities

## Abstract

**Background:**

Gender disparities in academic medicine persist, particularly in male-dominated fields such as orthopedic surgery. Social media platforms are reshaping academic communication, although data describing gender differences in use and engagement are limited.

**Objective:**

This study aims to examine gender differences by X (formerly known as Twitter) use among orthopedic surgeons, including variations in engagement, content, and influence.

**Methods:**

This cross-sectional study evaluated publicly available data from the 2023 US News and World Report top 20 hospitals for orthopedic surgery. Demographic data, apparent gender (binary), and public X data were collected.

**Results:**

Of 1327 orthopedic surgeons, 25% (332/1327) were on X. X users were more likely to hold leadership roles (*P*<.001), higher faculty appointments (*P*<.001), and additional advanced degrees (*P*=.007). Women X users (vs men) were less likely to be full professors (12% vs 20%; *P*=.04). While women (vs men) had similar numbers of followers, following, and posts (*P*>.05), women liked more posts (median 242 vs 35, *P*=.006). On thematic analysis of biographical content, women were more likely to mention being a parent, spouse, or their hobbies and interests (24.4% vs 12.1%; *P*=.048).

**Conclusions:**

Orthopedic surgeons on X were more likely to have higher academic rank, leadership titles, and dual degrees, although gender disparities persisted with women X users harboring lower rates of full professorship. Women orthopedic surgeons were more actively engaged with other posts on X. The motivation behind these trends is worthy of further study.

## Introduction

Women comprise more than half of medical students since 2019, but gender differences in promotion, tenure, and satisfaction in academic medicine persist [1]. According to the 2024 Association of American Medical Colleges report, women comprise 52% of medical graduates, 29% of full professors, and only 25% of department chairs [2]. These differences are particularly pronounced in orthopedic surgery, where women comprise 18% of the academic workforce (the lowest amongst all medical specialties), but hold only 9% of higher professorial ranks (including one department chair in 2016), and lag behind other specialties in increasing representation of women [3]. This dearth of women in top academic positions suggests a leaky pipeline, which in most cases begins after training and declines with each promotion.

Social media, particularly the platform X (formerly known as Twitter), is being increasingly used in professional settings. Relevant hashtags and retweets connect geographically distant individuals, enabling greater dissemination of research, enhancing digital scholarship, and building relationships [4-6]. Among orthopedists, hashtags such as #OrthoTwitter and #womeninortho have been developed to facilitate a social media community for education, networking, and professional development [7-9]. However, despite the professional popularity of X, there is a paucity of data on X usage and whether gender disparities persist in social media orthopedic surgery communities (such as X). Given the underrepresentation of women in orthopedic surgery, coupled with social media’s expanding professional reach, studies examining gender disparities in social media use and the workforce are essential. This study aims to examine gender differences by X use among orthopedic surgeons, as well as gender differences in X engagement, content, and influence.

## Methods

### Data Acquisition

The 20 best hospitals for orthopedic surgery were identified according to the 2023 US News and World Report rankings. Institutional websites and related faculty profiles were accessed from February 2024 to June 2024 to identify residency-trained orthopedic surgeons. Nonoperative sports medicine practitioners were excluded. Available faculty demographic data were collected, including geographical region, training dates, degrees received, and faculty appointments. Apparent gender (binary) was assigned using name, pronouns, and public profile images. For X information, publicly available data (eg, number of followers, following, likes, posts, account creation date, and biography) were manually extracted. Personal profiles and private accounts were excluded. Data was collected by a single investigator (JOG).

### Data Analysis

Length of training was defined as years of training since medical school graduation. To assess the thematic content of X profile biographies, text was classified based on mentioning one or more themes, including job roles, specialty, and personal information, which included mentions of parent, spouse, or personal interests. The characteristics of orthopedic surgeons were assessed based on X use and by gender among X users. Descriptive statistics included the Wilcoxon rank-sum test for continuous variables and the chi-square or Fisher exact test for categorical variables. Statistical analyses were performed using StataSE (version 17.0; StataCorp) with 2-sided tests at a significance level of .05.

### Ethical Considerations

This cross-sectional study evaluated publicly available data and was exempt from ethical approval according to the Cedars-Sinai Medical Center institutional review board (STUDY00003292). The STROBE (Strengthening the Reporting of Observational Studies in Epidemiology) reporting guidelines were followed.

## Results

A total of 1327 orthopedic surgeon profiles were analyzed, of which 330 (24.9%) were on X (Table 1). Compared with nonusers, X users were more likely to be geographically located in the Northeast (148/330, 44.9% vs 364/997, 36.5%; *P*<.001), hold leadership titles (*P*<.001), higher faculty appointments (*P*<.001), additional advanced degrees (*P*=.007), and have fewer years in practice since medical school completion (*P*=.02). Among X users, women (vs men) were less likely to be full professors (5/41, 12.2% vs 57/289, 19.7%; *P*=.04), although there was no significant difference in geographic location, leadership titles, or dual degree status (*P*>.05; Table 2).

While women (vs men) had similar numbers of followers, following, and posts (*P*>.05), women had higher levels of self-engagement in the form of liking more posts (median 242 vs 35, *P*=.006). On thematic analysis of X profile biographies, women (vs men) were more likely to mention personal information including being a parent, spouse, hobbies, or interests (10/41, 24.4% vs 35/289, 12.1%; *P*=.048), while men (vs women) were more likely to mention their job or specialty (270/289, 93.4% vs 34/41, 82.9%; *P*=.02).

**Table 1:** Baseline characteristics stratified by X use.

Variables		Not on X (n=997)	On X (n=330)	*P* value^a^	
**Geographic region, n (%)**					<.001
	Northeast	364 (36.51)	148 (44.85)		
	Midwest	178 (17.85)	75 (22.73)		
	South	84 (8.43)	30 (9.09)		
	West	371 (37.21)	77 (23.33)		
**Gender, n (%)**					.13
	Male	902 (90.47)	289 (87.58)		
	Female	95 (9.53)	41 (12.42)		
**Faculty type, n (%)**					<.001
	None or not listed	431 (43.23)	72 (21.82)		
	Instructor	40 (4.01)	12 (3.64)		
	Assistant	264 (26.48)	118 (35.76)		
	Associate	114 (11.43)	66 (20.00)		
	Full professor	148 (14.84)	62 (18.79)		
**Number of leadership titles, n (%)**					<.001
	0	731 (73.32)	184 (55.76)		
	1	182 (18.25)	91 (27.58)		
	2	57 (5.72)	37 (11.21)		
	More than 3	27 (2.71)	18 (5.45)		
**Subspecialty, n (%)**					<.001
	General	27 (2.71)	2 (0.61)		
	Sports medicine	205 (20.56)	106 (32.12)		
	Upper extremity	167 (16.75)	40 (12.12)		
	Lower extremity	259 (25.98)	74 (22.42)		
	Spine	159 (15.95)	33 (10.00)		
	Trauma	48 (4.81)	12 (3.64)		
	Pediatrics	43 (4.31)	18 (5.45)		
	Musculoskeletal oncology	20 (2.01)	10 (3.03)		
	Multiple and other	69 (6.92)	35 (10.61)		
**Dual degree (PhD, MBA, MPH, and MS), n (%)**					.007
	Yes	83 (8.32)	44 (13.33)		
	No	914 (91.68)	286 (86.67)		
Interval sine medical school (years), median (IQR)		24 (14-35)	21 (15-29)	.02	

^a^*P* value is calculated by the Wilcoxon rank-sum test for continuous variables and the chi-square test or Fisher exact test for categorical variables as appropriate.

^b^Not available.

**Table 2:** Characteristics of orthopedic surgeons on X stratified by gender.

Variables				Male (n=289)		Female (n=41)		*P* value^a^	
**Region, n (%)**									.37
		Northeast	126 (43.6)		22 (53.66)				
		Midwest	67 (23.18)		8 (19.51)				
		South	29 (10.03)		1 (2.44)				
		West	67 (23.18)		10 (24.39)				
**Faculty type, n (%)**									.04
		None or not listed	68 (23.53)		4 (9.76)				
		Instructor	12 (4.15)		0 (0)				
		Assistant	98 (33.91)		20 (48.78)				
		Associate	54 (18.69)		12 (29.27)				
		Full professor	57 (19.72)		5 (12.2)				
**Number of leadership titles, n (%)**									.95
		0	162 (56.06)		22 (53.66)				
		1	78 (26.99)		13 (31.71)				
		2	33 (11.42)		4 (9.76)				
		More than 3	16 (5.54)		2 (4.88)				
**Subspecialty, n (%)**									.005
		General	2 (0.69)		0 (0)				
		Sports medicine	94 (32.53)		12 (29.27)				
		Upper extremity	32 (11.07)		8 (19.51)				
		Lower extremity	69 (23.88)		5 (12.2)				
		Spine	32 (11.07)		1 (2.44)				
		Trauma	11 (3.81)		1 (2.44)				
		Pediatric	10 (3.46)		8 (19.51)				
		Musculoskeletal oncology	8 (2.77)		2 (4.88)				
		General	31 (10.73)		4 (9.76)				
**Dual degree (PhD, MBA, MPH, and MS), n (%)**									.45
		Yes	37 (12.8)		7 (17.07)				
		No	252 (87.2)		34 (82.93)				
Interval since graduating from medical school (years), median (IQR)				22 (16-30)		17 (12-24)		.002	
**X use variables (publicly available)**									
	Time on X (years), median (IQR)			9 (6-12)		8 (5-11)		.13	
	Average number of followers on X, median (IQR)			221 (40-717)		270 (79-773)		.39	
	Average following on X, median (IQR)			123 (31-307)		159 (32-426)		.41	
	Average number of liked posts on X, median (IQR)			35 (0-537.5)		242 (20-1110)		.006	
	Average number of posts on X, median (IQR)			103 (18-579.5)		83 (17-452)		.71	
**Thematic content of X profile biography, n (%)**									.02
	Job roles and specialty			270 (93.43)		34 (82.93)			
	Mention			19 (6.57)		7 (17.07)			
	No mention			—^b^		—			
**Personal information (parent, spouse, and interests), n (%)**									.048
	Mention			35 (12.11)		10 (24.39)			
	No mention			254 (87.89)		31 (75.61)			

^a^*P* value is calculated by the Wilcoxon rank-sum test for continuous variables and the chi-square test or Fisher exact test for categorical variables as appropriate.

^b^Not available.

## Discussion

### Principal Findings

In this cross-sectional study, we explored demographic and professional characteristics of X use by gender in the social media orthopedic surgery workforce. The strength of this study is the robust analysis of more than 1000 orthopedic surgeons, including detailed evaluations of more than 300 X profiles and their usage statistics. We observed that while X users (vs non-X users) were more likely to hold leadership titles and higher faculty appointments, women X users (vs men X users) were less likely to be full professors, highlighting the persistence of gender disparities among orthopedic surgeons on X. Furthermore, women orthopedic surgeon X users demonstrated greater engagement (in the form of interacting with posts), suggestive of gender-based differences in motivation for professional X use, which is worthy of further study.

### Comparisons With Previous Work

In 2021, Friedman and Menendez [10] highlighted the use of #OrthoTwitter on social media to connect the orthopedic community amidst isolation during the COVID-19 pandemic. In the same year, Cole et al [11] concluded that social media is a powerful tool in orthopedic surgery resident training by enhancing worldwide communication. This is consistent with the rise in popularity of professional X use among orthopedic surgeons, with nearly 32,000 posts and more than 700,000 likes using #OrthoTwitter in 2022 [8]. In a 2023 study evaluating the use of social media by women surgeons, the authors reported that the majority of posts were for personal reasons (44%) and suggested the use of the hashtag #womeninortho to maximize the reach of their content [7]. Building upon these findings, we observed that women were more likely to engage with posts from other users and share personal or family information in their profile biographies, consistent with studies showing that women physicians are more likely than men to use social media to build support networks and foster communities [12].

In a scoping review of the demographic characteristics of X influencers in academic medicine, Istl et al [13] analyzed 13 studies and described persistent gender disparities on X in academic medicine. This study builds upon these findings, focusing on the male-dominated specialty of orthopedic surgery, which lags behind other specialties in increasing the representation of women [3]. We observed that despite X users (vs nonusers) being enriched for more leadership titles, higher faculty appointments, and dual advanced degrees, women orthopedic surgeon X users (vs men), remained less likely to be full professors. The various potential reasons for disparate ascension in academic medicine, particularly for surgeons, are several-fold. Women physicians are more likely to take on greater parenting roles, sacrifice work time for domestic responsibilities, and have spouses who are working full-time [14]. Women surgeons face disproportionate conflicts of work-life integration and threats to their careers from pregnancy and childbearing [15,16], and for physician mothers in procedural fields, these self-reported greater familial demands are associated with lower overall career satisfaction [17,18]. Further, women physicians face gender discrimination and sexual harassment, manifesting as workplace microaggressions, lower rates of promotion, and unequal compensation [16,19,20]. Each of these factors may limit career progression and promote the early exit of women physicians.

Some have proposed that social media platforms such as X would offer women an equal opportunity to share their perspectives, promote their work, and connect with same-gender role models [4], the latter of which has been identified as a most significant barrier faced by female professionals [21-24]. The results of our study, however, suggest that this leveling is not realized in practice. We observed similar levels of X followers and posts between men and women, aligning with survey data demonstrating that men and women physicians report similar rates of using social media to build their professional network and increase collaborations [12]. However, despite similar intent for professional development, women physicians are less likely to report career-advancing benefits (ie, speaking engagement or expanded research portfolio) [12]. Further, others have shown that despite similar follow count, men physicians are more likely to hold a “verified” X account, a (now controversial) designation of validity [25]. Together, these data underscore the need for further research to understand motivational and behavioral differences in professional social media use and identify strategies to improve gender inequities.

### Limitations

This study has several potential limitations, including inaccuracies or incompleteness in publicly available data. The use of binary gender classification may introduce classification bias or inaccuracy in the preferred gender. In addition, only physicians from top US News and World Report hospitals were selected, which may not reflect other medical practice settings. In addition, the cross-sectional design limits causal inference, for instance, whether X users were more likely to hold leadership roles or higher faculty appointments due to social media presence or whether these positions contributed to social media presence or use, which is worthy of further study.

### Conclusions

Gender disparities persist in the orthopedic surgery X community, although women orthopedic surgeons were more actively engaged. The motivation behind these trends, the temporal relationship between social media use and leadership positions, and the impact of social media use on gender disparities in professional development are worthy of further study.

## Abbreviations

STROBE: Strengthening the Reporting of Observational Studies in Epidemiology
